# Pancreatic Ductal Adenocarcinoma and Nutrition: Exploring the Role of Diet and Gut Health

**DOI:** 10.3390/nu15204465

**Published:** 2023-10-21

**Authors:** Paola Gualtieri, Rossella Cianci, Giulia Frank, Erica Pizzocaro, Gemma Lou De Santis, Silvia Giannattasio, Giuseppe Merra, Giovanni Butturini, Antonino De Lorenzo, Laura Di Renzo

**Affiliations:** 1 Section of Clinical Nutrition and Nutrigenomics, Department of Biomedicine and Prevention, University of Rome Tor Vergata, Via Montpellier 1, 00133 Rome, Italygiuseppe.merra@uniroma2.it (G.M.); laura.di.renzo@uniroma2.it (L.D.R.); 2 Department of Translational Medicine and Surgery, Catholic University of the Sacred Heart, Fondazione Policlinico Universitario “A. Gemelli”, IRCCS, Largo A. Gemelli 8, 00168 Rome, Italy; 3 School of Specialization in Food Science, University of Tor Vergata, Via Montpellier 1, 00133 Rome, Italy; giulia.frank@ymail.com (G.F.); ericapizzocaro@gmail.com (E.P.); silviagiannattasio85@gmail.com (S.G.); 4 PhD School of Applied Medical-Surgical Sciences, University of Rome Tor Vergata, Via Montpellier 1, 00133 Rome, Italy; gemmaloudesantis@gmail.com; 5 Division of Hepato-Bilio-Pancreatic Surgery, P. Pederzoli Hospital, Via Monte Baldo 24, 37019 Peschiera del Garda, Italy; butturinichirurgo@gmail.com

**Keywords:** pancreatic ductal adenocarcinoma, immunonutrition, gut microbiota, dysbiosis, chronic inflammation

## Abstract

The incidence of pancreatic cancer is increasing worldwide. The most common form is represented by pancreatic ductal adenocarcinoma (PDAC) which has been shown to be linked to chronic inflammation. Notably, the gut microbiota has emerged as a critical player in regulating immune responses and inflammation. Indeed, intestinal dysbiosis, characterized by an imbalance in the gut microbiota composition, can contribute to the initiation of chronic inflammation. Sterile chronic inflammation can occur, probably activated by the translocation of bacterial components, such as lipopolysaccharide (LPS), the major component of Gram-negative microbiota, with the consequent induction of innate mucosal immunity, through the activation of Toll-like receptors (TLRs). Furthermore, the interaction between LPS and TLRs could enhance cancer progression. Recent research has shed light on the pivotal role of nutrition, as a modifiable risk factor, in PDAC immunological processes, particularly focusing on the immuno-modulatory effects of the gut microbiota. Different dietary regimens, fiber intake, immunonutrients, and antioxidants have the potential to either exacerbate or mitigate chronic inflammation, thereby influencing the pathogenesis and natural history of PDAC. These dietary components may affect the gut microbiota composition and, consequently, the level of inflammation, either promoting or protecting against PDAC. In this review of reviews, we discuss the modulatory role of nutrition and the gut microbiota in PDAC’s immunological processes to explore a translational therapeutic approach that could improve the survival and quality of life of these patients.

## 1. Introduction

The incidence of pancreatic cancer is increasing worldwide [1]. Pancreatic ductal adenocarcinoma (PDAC) represents about 90% of pancreatic cancers and affects the exocrine cells of the pancreas, which produce enzymes and digestive juices [2]. It is the most difficult cancer to treat, and it has a survival rate of 9% at 5 years. Nearly 500,00 new cases are diagnosed and over 450,000 people die from PDAC yearly [3,4]. PDAC data-based projections in the United States suggest that it could become a leading cause of death in the next decade regardless of cancer type [5,6]. Regarding symptoms, which occur with a late onset, jaundice is the only one with a solid predictive link to pancreatic cancer, while several nonspecific symptoms such as vomiting, nausea, back pain, abdominal pain, diarrhea, constipation, malaise, and weight loss have a weaker association [7]. So, unfortunately, pancreatic cancer is diagnosed at an advanced stage. Only 10–15% of new cases of PDAC diagnosed at an early stage are resectable; in these cases, the prognosis for life is much better, and could lead to a higher probability of survival, with an average rate of up to 25% [8].

The PDAC markers that are analyzed are detectable in the pancreatic tissue, urine, serum, and feces. To date, serum carbohydrate antigen (CA) 19-9 is the only approved biomarker of PDAC [1]. CA 19-9 is used as a marker for the monitoring and surveillance of PDAC progression. CA 19-9 is associated with cancer cells, but has limited specificity for this type of cancer; therefore, it cannot be used for diagnostic purposes [9].

Other biomarkers for PDAC are represented by proteins, autoantibodies, circulating DNA, microRNA, methylated DNA, exosomes [10]. DNA mutations have been detected in the pancreatic juice of patients who later developed invasive cancers. Particularly, the presence of the P53 mutant has been identified in individuals with PanIN 2–3, intermediate- and high-grade IPMN, and invasive malignancies [11]. Although PDAC exhibits a high rate of genetic mutations, like the Kirsten rat sarcoma virus (KRAS), the use of circulating tumor cells or circulating tumor DNA is not suitable for PDAC screening or diagnosis due to their limited sensitivity [10]. Instead, non-invasive early detection biomarkers have been explored, such as the increased concentration of specific volatile organic compounds (VOCs) in exhaled air, with good sensitivity (100%) and specificity (84%). Particularly, some molecules were identified for the PDAC model, such as ammonia, sulfur dioxide, hydrogen sulfide, acetyl group and acetaldehyde [12].

Given the late onset of symptoms, the strategy to achieve early diagnosis is to identify and closely monitor patients with cancer-associated risk factors, modifiable and nonmodifiable. Particularly, modifiable risk factors are represented by smoking and alcohol abuse, chronic pancreatitis, obesity, dietary factors, and infections. Nonmodifiable risk factors are age, sex, ethnicity, gut microbiota, and genetics [13].

The most largely modifiable risk factor is represented by diet; exploring and understanding dietary risk factors are crucial. Primary and secondary preventions are fundamental [14]. Indeed, dietary intervention could be helpful in controlling blood glucose and insulin levels, as well as in reducing abdominal fat, associated with an increased PDAC risk, in overweight and obese individuals [15]. Several studies have shown that obesity and type II diabetes mellitus lead to PDAC development and progression causing a chronic, systemic, low-grade inflammatory condition [16]. An excessive caloric intake due to an increase in dietary fat and red meat leads to abnormal lipid metabolism and promotes the development of reactive oxygen species (ROS), while a high intake of soft drinks containing sugar and fructose results in a chronic increase in insulin levels [16]. However, Milajerdi et al. [17] did not observe any significant association between sugar-sweetened beverage consumption and PDAC risk in adults. On the other hand, the consumption of sugar-sweetened beverages leads to a higher total energy intake and, consequently, to a higher risk of becoming overweight and obese [17]. Although there is a clear link between increased body fat and the PDAC risk, it is still unclear whether diets rich in omega-3 (Ω-3) polyunsaturated fatty acids are associated with a reduced PDAC risk and whether, in general, saturated, monounsaturated, or polyunsaturated fatty acids may influence PDAC onset and progression [18].

Particularly, it is known that a greater adherence to the Mediterranean diet can reduce the risk of PDAC, particularly in women compared to men [19].

Furthermore, it is well known how diet can affect the gut microbiota [20,21]. Higher levels of *Granulicatella adiacens* and *Porphyromonas gingivalis*, and lower levels of *Streptococcus mitis* and *Neisseria elongata* have been observed in PDAC patients, thus correlating with an increased risk of pancreatic cancer [22]. However, it is currently unknown whether targeted treatment of the gut microbiota is a therapeutic option and diet can be a viable intervention tool.

The aim of this review of reviews [23] is to evaluate the recent evidence on the modulatory role of nutrition in PDAC immunological processes, including the immunomodulatory role of the gut microbiota.

## 2. Materials and Methods

The literature search was conducted in April 2023 via scientific databases available online (Pubmed, EBASE, Web of Science, Google Scholar and Cochrane Library) and focused on reviews, systematic reviews, and meta-analyses. Relevant keywords to the terms “pancreatic cancer” OR “pancreas cancer” OR “pancreatic carcinoma” were analyzed in association with “immunology” OR “gut microbiota” OR “nutrition” OR “nutrients” using the AND modifier. Only reviews published in peer-reviewed journals were considered to meet our inclusion criteria: articles including specific references to pancreatic cancer and immunology, gut microbiota, or nutrition either in the title, the abstract, or the text. Original and primary studies and surgery treatment-related studies were excluded. Six different operators independently performed the search, according to the Preferred Reporting Items for Systematic Reviews and Meta-Analyses (PRISMA) scheme and the REAPPRAISED checklist [24]. The selection process was carried out by first analyzing the titles, then the abstracts, and finally, the full text. A check of article titles was made, and duplicates were excluded. Overall, 251 articles were identified, of which 37 were duplicates, 54 were excluded after two reviewers’ provisional assessment of titles and abstracts and 121 were excluded after full-text screening. Finally, 39 articles relevant to the topic were found, including 32 reviews, 1 mini-review, 3 systematic reviews, and 3 meta-analyses.

**Figure 1:**
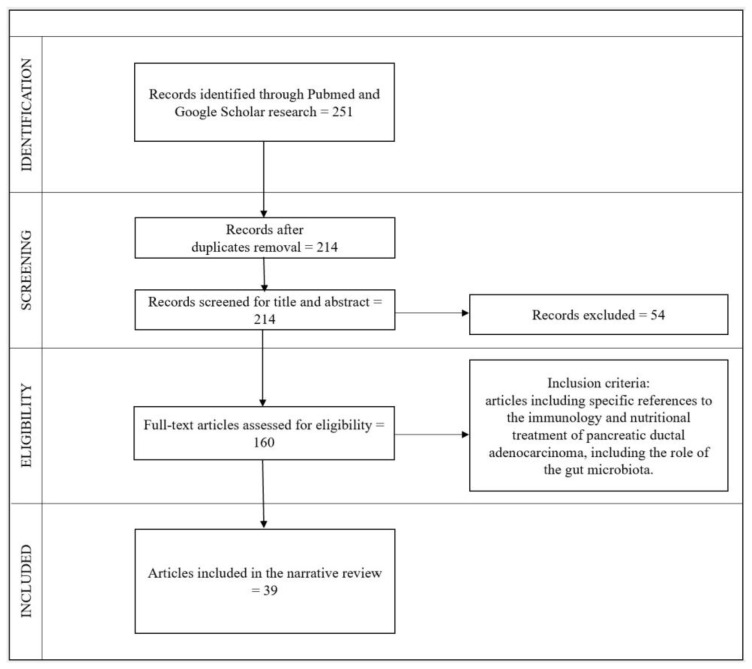
Flow diagram of study selection.

The selection, inclusion, and analysis process are illustrated in Figure 1.

## 3. PDAC Immunology

In recent decades, it has been well-established that PDAC is linked to chronic inflammation [25,26,27]. Indeed, an increased risk of cancer is present in patients with chronic pancreatitis associated with Kirsten rat sarcoma virus (KRAS) oncogene mutation [28]. To this purpose, the pivotal role of intestinal dysbiosis in triggering the immune system response through the innate immunity arm, involving Toll-like receptors (TLRs), tumor-infiltrating lymphocytes (TILs), and proinflammatory mediators, which are able to induce cancerogenic pathways, has been considered [29,30]. The innate immune system is composed of macrophages, granulocytes, mast cells, dendritic (DC) cells, myeloid-derived suppressor cells (MDSC), and natural killers (NK). After maturation, macrophages are divided into M1 (pro-inflammatory) and M2 (anti-inflammatory) cells; these latter can favor cancer progression [31]. TLRs are a part of innate immunity. TLR signaling has been shown to be central in pancreatic cancer. It has been demonstrated that sterile chronic inflammation is related to PDAC, probably activated by the translocation of bacterial components such as lipopolysaccharide (LPS), the major component of Gram-negative microbiota, with consequent induction of TLRs, such as TLR2, TLR4, and TLR9 [32]. The interaction between LPS and TLR4 seems to be able to induce cancer progression [33]. Moreover, inflammatory and carcinogenetic actions consequent to the inhibition of the TLR adapter protein myeloid differentiation primary response gene 88 (MyD88) are linked to dendritic cell-mediated antigen restricted Th2 cells [34]. Another type of innate immune cell implicated in pancreatic cell growth and invasion are the mast cells, which show angiogenetic effects and can upregulate regulatory T-lymphocytes (Tregs) in a cytokine-dependent manner [35]. Moreover, MDSCs play a certain role in suppressing immune responses against cancer cells and in enhancing immune evasion [36]. In PDAC, there is a large amount of MDSCs with respect to healthy controls and this is related to higher levels of Th2 CD4 cells and Tregs [37].

At the stromal tissue level, the tumor environment is characterized by the continuous cross-talk between the immune system, cancer cells, several pro-inflammatory (such as, IL-1β, IL-2, IL-6, IL-17, and TNFα) and anti-inflammatory cytokines (such as TGFβ and IL-10) [38], chemokines, and chemokine ligands (such as chemokine ligand 2 (CCL2) and C–X–C motif chemokine ligand 12 (CXCL12)) [39]. Moreover, the PDAC microenvironment is characterized by a low number of anti-tumor T-cells, and a high rate of M2 macrophages, MDSCs, and Tregs [40]. Furthermore, Tregs are activated by MDSCs in a cytokine-independent manner and induce the suppression of T-lymphocytes. The presence of Treg cells at the tumor tissue level represents a bad prognostic factor for clinical outcome. Tregs can downregulate the anticancer activity of effector T lymphocytes and NKs and release cytokines, such as TGFβ and IL-10, thus creating an anti-inflammatory environment [41].

Recently, oncogenic short non-coding RNA sequences that regulate mRNA translation, the microRNAs (miRNA), have been shown to be associated with the progression of pancreatic cancer [42] and to be influenced by, and, at the same time, influence different cytokines and other mediators of inflammation [43].

The characteristics of all the articles on PDAC immunology are listed in Table 1.

## 4. PDAC and Gut Microbiota

In PDAC patients, a condition of dysbiosis characterized by reduced microbial diversity and a large amount of LPS-producing bacteria has been shown. In particular, these pathogens determine oxidative damage via the NF-kB pathway and related cytokines [7]. For example, *Helicobacter pylori* seems to be involved in PDAC onset through the activation of the NF-κB pathway, the activator of signal transducer and activator of transcription3 (STAT3); furthermore, its LPS prompts KRAS oncogene mutation [44].

Multiple microbial species from fecal and oral samples have demonstrated potential as diagnostic markers for PDAC, showing associations with the CA 19-9 marker [45]. These include increased levels of *Streptococcus* and *Veillonella* and decreased levels of *Faecalibacterium prausnitzii* [46].

While the five-year survival rate for PDAC patients is typically quite low, a microbial signature has been identified in individuals who managed to survive beyond this critical period. This signature is characterized by increased species diversity of microorganisms such as *Pseudoxanthomonas*, *Streptomyces*, *Saccharopolyspora*, and *Bacillus clausii*. Additionally, studies have revealed that transplanting the fecal microbiota from these long-term PDAC survivors into mice could influence tumor growth [47].

Indeed, a strong connection exists between the gut microbiota and PDAC, and emerging therapeutic approaches include supplementing with prebiotics, both traditional and new-generation probiotics, synbiotics, and fecal microbiota transplantation. In one study, evaluating oral *Neisseria elongata* and *Streptococcus mitis* allowed for the differentiation of PDAC patients from healthy individuals with high sensitivity (96.4%) and specificity (82.1%). Furthermore, an elevated presence of *Fusobacterium* within the intra-tumor microenvironment may serve as a cancer marker, in contrast to the reduced levels of *Lactobacilli* in healthy controls [48].

Furthermore, PDAC patients exhibited an increase in Proteobacteria, *Klebsiella pneumoniae, Clostridium bolteae, Clostridium symbiosum, Streptococcus mutans, Alistipes shahii,* four species of Bacteroidetes, and two of Parabacteroides, along with a reduction in the Firmicutes phylum. There was also a higher presence of fecal *Bifidobacterium animalis, Collinsella aerofaciens, Eubacterium ventriosum, Klebsiella pneumoniae, Roseburia intestinalis,* and *Streptococcus thermophilus*, which may serve as a prognostic indicator for the disease. Additionally, PDAC patients exhibited lower levels of butyrate-producing bacteria. Given these variations in microbial composition between early and advanced cancer patients compared to healthy individuals, microbial dysbiosis holds promise as a potential diagnostic and prognostic marker for this disease [45].

Moreover, PDAC patients who received synbiotics—a combination of probiotics (*Enterococcus faecalis*, *Clostridium butyricum,* and *Bacillus mesentericus*) and prebiotics containing inulin, beta-glucan, resistant starch, and pectin—exhibited a reduced occurrence of systemic response syndrome, late organ failure, multiple organ failure, septic complications, and mortality. While the research on the role of postbiotics in PDAC patients is limited, it is hypothesized that their mechanisms of action are likely related to their anti-inflammatory properties and their ability to restore intestinal barrier integrity, rather than exerting a direct cytotoxic effect on the tumor [48].

The characteristics of all the articles on PDAC and gut microbiota are listed in Table 2.

## 5. Role of Nutrition and PDAC

PDAC patients commonly experience cachexia, malnutrition, weight loss, and reduced lean mass, often accompanied by systemic inflammation. At the time of diagnosis, patients frequently exhibit a significant 10% weight loss, which substantially impairs their quality of life. These conditions, including malnutrition, cachexia, and weight loss, are complex and influenced by various factors, such as the release of pro-inflammatory cytokines, heightened lipolysis and glycolysis, pancreatic duct obstruction, and issues related to gut microbiota dysbiosis, particularly following prior pancreatitis.

Several nutritional strategies have been employed to counteract cachexia and malnutrition in PDAC patients, including methods like parenteral and enteral nutrition or dietary supplements [49]. Nutritional assessment and subsequent interventions, such as the utilization of the Malnutrition Universal Screening Tool (MUST), help gauge the level of risk associated with weight loss and disease severity. This information aids in determining whether parenteral, enteral, or oral supplementation is necessary. More recently, pancreatic enzyme replacement therapy (PERT) has become a component of nutritional management for these patients, even after pancreatectomy, contributing to a swift recovery from weight loss [50].

Cañamares-Orbís et al. [50] evaluated the role of Medical Nutrition Therapy (MNT) as part of the management of pancreatic cancer, patients with which should undergo constant nutritional therapy to optimize clinical outcomes. Early nutritional intervention, involving both screening and treatment, decreases morbidity, reduces hospital stays, and lowers hospitalization costs for inpatients. In cases of pancreatic cancer where patients cannot meet 100% of their daily nutritional needs through diet alone, oral nutritional supplements (ONS) are advised. If ONS are still insufficient, additional support through enteral or parenteral nutrition may be required. Furthermore, individuals at risk of malnutrition should receive ONS for a minimum of 5–7 days prior to surgery. As a preoperative measure, patients scheduled for pancreatectomy should be incorporated into the ERAS (Enhanced Recovery After Surgery) protocol, which entails reducing the preoperative fasting period (6 h for solids and 2 h for liquids) and providing oral carbohydrate loading 2 h before surgery for non-diabetic patients. It is important to note that despite extensive research efforts, there is currently no established standard of care or approved pharmaceutical treatment for cachexia associated with PDAC [50,51].

Ketogenic diets (KDs) have gained attention for their potential in fighting cancer, including pancreatic cancer, as they exhibit anti-tumor and anti-inflammatory properties. However, there is limited evidence on how KDs specifically affect pancreatic cancer-associated cachexia. KDs lack a standardized composition, with varying levels of fat, protein, and carbohydrates, leading to different outcomes. Currently, KDs appear to help preserve muscle mass, which could potentially reduce cachexia development. Nonetheless, long-term KDs can be challenging to sustain due to side effects like constipation, diarrhea, and fatigue, posing a risk of malnutrition, especially for pancreatic cancer patients. Furthermore, pancreatic exocrine insufficiency (PEI), common in PDAC, contributes to cachexia due to impaired fat absorption and is linked to poor survival in advanced PDAC patients. Since PEI is prevalent in pancreatic cancer, patients usually undergo pancreatic enzyme replacement therapy (PERT). Consequently, a high-fat KD may not be a suitable dietary strategy in this context [52].

Thus far, two distinct dietary approaches warrant further investigation. The first approach involves an alkaline diet, emphasizing a minimum daily intake of 400 g of fruits and vegetables while excluding meat and dairy products. Patients were also recommended an oral bicarbonate supplement of 3 to 5 g per day if their urine pH fell below 7, with reported survival improvements in advanced-stage patients whose urine pH ≥ 7. The second dietary regimen combines the consumption of raw or lightly steamed fruits and vegetables, regular vegetable juicing, vegetable protein, daily yogurt, one–two eggs weekly, fish two–three times per week, and excludes meat. It additionally includes supplements of vitamins, minerals, trace elements, freeze-dried thyme, liver supplements, porcine lyophilized pancreas, and detoxification products. This intervention resulted in a notable 1-year survival rate, with 81% of patients surviving, demonstrating a 25% survival rate across all stages of pancreatic cancer [51].

Additionally, the consumption of dietary fiber, which comprises a group of edible organic compounds resistant to digestion and absorption in the human small intestine, is recommended. One study indicated that an increased intake of fiber as part of a healthy diet had a beneficial effect on PDAC [53].

To assess the inflammatory properties of a diet, researchers developed the Dietary Inflammatory Index (DII). This index categorizes foods into various groups and assigns them specific scores to evaluate the overall inflammatory impact of the diet. A high DII score, reflecting a diet with heightened inflammatory qualities, is linked to an increased risk of PDAC [54]. DII confers a more comprehensive understanding than studying a single food or nutrient. Indeed, it considers both anti-inflammatory components and includes six pro-inflammatory biomarkers (C-reactive protein (CRP), TNF-α, IL-1, IL-4, IL-6, and IL-10), along with factors like cholesterol, total fat, saturated fat, trans-fatty acids, protein, iron, vitamin B12, carbohydrates, and energy intake [55].

### 5.1. Immunonutrients and PDAC

In PDAC patients’ nutritional therapy, several studies on oral supplements, including micronutrients such as ω-3, vitamins (i.e., A, D, E, K, and B12), and minerals (i.e., iron, zinc, and selenium), in parallel with the continuous monitoring of a food diary and lifestyle, have been conducted [50].

Ω-3s are frequently analyzed in PDAC not only for their ever-present deficiency but also as a preventive therapy. Ω-3 deficiency, typical of Western diets, increases the risk of developing tumors, especially in the gastrointestinal tract, due to the altered composition of cell membranes as a result of an imbalance in the synthesis of lipids and cholesterol in newly formed tissue. Transgenic animals massively producing ω-3 showed a reduced risk of developing *Helicobacter pylori*-related cancer, polyposis, and colitis. However, further studies are needed to investigate the action of ω-3 in the prevention and treatment of PDAC patients [56]. No effect on body composition and no change in albumin and CRP values related to ω-3 consumption were observed. However, a mixture of ω-3 fatty acids and amino acids, such as glutamine and arginine, antioxidants, and nucleotides reduced hospitalization and post-operative complications [51].

Furthermore, the daily consumption of 10 mg/day of vitamin B12 or D can drastically reduce the PDAC incidence by 27% for vitamin B12 and 25% for vitamin D, whereas the intake of other vitamins, both antioxidant and non-antioxidant, did not demonstrate the same results [57].

### 5.2. Antioxidants and PDAC

Antioxidants are responsible for detoxifying and blocking ROS development to prevent DNA damage, avoiding mechanisms that lead to tumor onset and progression.

In this context, an antioxidant-rich diet could be useful to contrast tumor progression. Several flavonoids, for example, have been analyzed to balance ROS production; they showed cardioprotective effects and beneficial properties against tumor progression and invasion.

Licorice is composed of more than 20 triterpenoids and 300 flavonoids, with the key active constituents: glycyrrhizin, glycyrrhetinic acid, licochalcone A, licochalcone E, glabridin, and liquiritigenin. In particular, glycyrrhizin has been studied for its oncotherapeutic and oncopreventive functions. In contrast, the excessive use of licorice could induce nephrotoxicity that can worsen hypertension and a mineral state unbalance.

Isoliquiritigenin (ISL) seems to have anti-inflammatory and anti-microbial effects through NF-kB and immune response regulation. In most human PDACs, the basal level of autophagy is elevated with ROS accumulation. In this context, it has been shown that ISL, in contrast to its normal activity, increased the ROS levels through the inhibition of autophagy in pancreatic cancer. On the other hand, pancreatic cancer with low levels of ROS is more resistant to chemotherapy [58]. At the same time, classical therapies can equally act on cancer cells, but prolonged therapies induce drug resistance. A combination of these approaches, ISL with several chemotherapeutic agents, could help to suppress pancreatic cancer cells.

Another important flavonoid, with anticancer properties, is quercetin. Quercetin presents several benefits on human health. It seems to exhibit a more metastasis-inhibiting effect than other molecules like, for example, resveratrol, probably down-regulating c-Myc expression and reducing TGF-α levels that contrast PDAC proliferation. It is also able to promote autophagy and apoptosis following mTOR blocking. Quercetin is ubiquitous in several vegetables, seeds, fruits, tea, and red wine [59].

The silymarin flavonoid, extracted from the seeds of *Sylibum marianum*, is a mixture of six flavolignan isomers. Among these compounds, silybin is functionally the most active. Koltai et al. highlighted its anticancer property on pancreatic cancer, alone or in combination with other molecules, like histone deacetylase inhibitors (HDAC). Silibinin is also able to regulate the glycolytic activity and proliferation of cancer cells and cachexia [60].

Another important antioxidant, which is not a flavonoid, is hydrogen sulfide (H_2_S). It is well-established that exogenous hydrogen sulfide (H_2_S) is essential for tumor suppression. Unfortunately, there are few pieces of evidence about H_2_S and PDAC. Moreover, it is also well-understood that some H_2_S donors, like phenethyl isothiocyanate (PEITC), slowly release H_2_S in the biological environment. The consequent higher concentrations of natural isothiocyanates (ITCs) are responsible for blocking tumor progression. Furthermore, PEITC and other H_2_S donors could boost chemotherapeutic agents [61].

### 5.3. Diet, Gut Microbiota and PDAC

Regarding the correlation between diet, dietary habits, and PDAC risk development and treatment, it is well established that a poor quality of diet and lifestyle is essential for PDAC occurrence, microbiota activity, and homeostasis.

In PDAC patients, there are some important changes in the gut microbiota composition and juice production. For example, a large consumption of alcohol could influence gut microbiota especially in *Verrucomicrobia*, *Actinobacteria*, and *Proteobacteria* families that were enriched. There is also a significant reduction in *Firmicutes* and *Lactobacillus*, while *Enterococcus* is significantly increased. Acetaldehyde, one of the important metabolites of ethanol, can promote inflammation, leading to chronic pancreatitis and then cancer [62].

Following macronutrients’ implication in PDAC, protein intake is associated with a significant risk of cancer. Ibrahim et al. [63] have tried to explain several correlations between certain types of meat and fish and PDAC, finding that proteins from refined grains, processed meat, and non-fried fish are considered risk factors for PDAC. On the contrary, nuts may have a protective role in PDAC.

Regarding to fats, it seems that a diet rich in saturated fatty acids (SFAs), monounsaturated fatty acids (MUFAs), and cholesterol are highly considered risk factors for PDAC. In contrast, polyunsaturated fatty acids (PUFAs) have a protective factor in PDAC.

Fat and water-soluble vitamins have a protective role in PDAC development because some group B vitamins, for example, are cofactors in DNA synthesis and are involved in methylation in one-carbon metabolism. Lower levels of vitamin B6 are associated with oncogenesis because they are associated with hypomethylation and DNA damage.

Diets enriched in red meat and/or sugar and sugar-sweetened soft drinks are more susceptible to help the progression of PDAC, also because these types of eating habits could alter the gut microbiota and lead to gut dysbiosis, which contributes to cancer progression. High meat consumption related to the Western diet increases *Bacteroides*, *Alistipes*, and *Bilophila* and decreases *Bifidobacterium*, *Roseburia*, *Eubacterium*, and *Ruminococcus*.

On the other hand, the beneficial effects of the Mediterranean diet (MD) seem to be effective to protect against PDAC development and progression, because the typically plant-based food composition seems to lead to a significant reduction in insulin and insulin-like growth factor (IGF-1), oestrogen, and testosterone; all of these can promote the development of PDAC.

The higher amount of fiber intake in MDs can eliminate carcinogens from the gut. The indirect effect is mediated by the modification of fiber to short-chain fatty acids (SCFAs) by gut microbiota metabolism [19].

The characteristics of all the articles on PDAC and nutrition are listed in Table 3.

## 6. Conclusions

Several nutritional strategies have been employed to address cachexia and malnutrition in PDAC patients, including parenteral and enteral nutrition or dietary supplements. Research also suggests that dietary antioxidants and specific compounds like quercetin, silymarin, and H_2_S donors, such as PEITC, may play a role in PDAC prevention and treatment by reducing ROS levels, inhibiting autophagy, and potentially enhancing the effectiveness of chemotherapeutic agents.

Additionally, diet significantly impacts PDAC development and treatment by modulating the immune response and affecting microbiota balance, particularly through nutrients like proteins, fat, and vitamins. Certain eating patterns, such as those rich in red meat and sugary drinks, can increase PDAC risk by altering gut microbiota and leading to gut dysbiosis. In contrast, maintaining a good quality of diet and correct lifestyle is crucial for preserving the microbiota diversity and homeostasis. Notably, MD exhibits protective effects due to its ability to eliminate carcinogens by promoting the production of short-chain fatty acids through the gut microbiota.

Particularly, gut microbiota is recognized to play a role in the onset and development of PDAC, and its manipulation presents a translational therapeutic approach for pancreatic cancers. These changes in the microbiota can influence cancer progression and metastasizing capacity. Moreover, microbiota can serve as a diagnostic tool in the future, and monitoring its health status through specific immunonutrition could improve patients’ prognosis.

Thus, a precision therapeutic approach can be particularly useful for predicting the effects of chemotherapy or immunotherapy on pancreatic cancer, and ongoing meta- and immuno-genomic studies hold promise for improving the survival and quality of life of PDAC patients.

## Figures and Tables

**Table 1:** Summary of characteristics of included studies on PDAC immunology in most recent reviews, systematic reviews, and meta-analyses.

Author	Title	Type of Paper	Date	Finding
Pagliari D. et al. [26]	Gut Microbiota-Immune System Crosstalk and Pancreatic Disorders	Review	2018	Chronic inflammation-related PDAC can arise independently of bacteria, driven by sterile inflammation triggered by intestinal dysbiosis and immune system activation through TLRs. The gut microbiota and antibiotics may affect chemotherapy responses and the tumor microenvironment, suggesting their role in treatment efficacy in PDAC.
Padoan A. et al. [27]	Inflammation and Pancreatic Cancer: Focus on Metabolism, Cytokines, and Immunity	Review	2019	PDAC risk is heightened by inflammation and ‘metaflammation’, yet PDAC triggers an immunosuppressive inflammatory response. The interplay between cytokines and chemokines produced by inflammatory cells and those produced by cancer plays a crucial role on both cancer development and inflammatory response. The key role of TNFα is highlighted.
di Magliano M.P. et al. [28]	Roles for KRAS in pancreatic tumor development and progression	Review	2013	Sustained KRAS activity is crucial for pancreatic tumorigenesis, and whether oncogenic KRAS expression alone generates the required activation levels or if additional upstream signals are necessary remains unclear. This uncertainty suggests potential avenues for pancreatic cancer prevention, which could involve minimizing factors that activate KRAS, such as addressing inflammation and lifestyle factors.
Cianci R. et al. [29]	Tissue infiltrating lymphocytes: the role of cytokines in their growth and differentiation	Review	2010	This review focuses on the role of local tissue cytokines in influencing T-cell proliferation and differentiation, highlighting the remarkable plasticity of CD4+ T-cells with numerous differentiation possibilities. The role of TILs and their microenvironment is discussed in various diseases, including tumors, along with an exploration of the role of apoptosis and the mucosal immune environment.
Cianci R. et al. [30]	The Interplay between Immunity and Microbiota at Intestinal Immunological Niche: The Case of Cancer	Review	2019	The microbiota exerts both immune-modulated and direct effects on the carcinogenesis of the gastrointestinal tract, including organs like the pancreas, not directly colonized by microbes. It plays a key role in cancer development, therapy response, and even the influences therapeutic strategies, highlighting the potential benefits of the gut microbiota modulation for both cancer patients and as a preventive approach for the general population.
Inman K.S. et al. [31]	Complex role for the immune system in initiation and progression of pancreatic cancer	Review	2014	In PDAC, there is a notable presence of various immunosuppressive cell types and a dysfunction of the immune response. This immune dysfunction involves the activation of immunosuppressive cells, the presence of immune cells that support tumor growth, and a deficiency in functional immune cells, ultimately reducing a vital barrier to tumor growth.
Orlacchio A. et al. [32]	The Role of Toll-like Receptors (TLRs) Mediated Inflammation in Pancreatic Cancer Pathophysiology	Review	2021	TLRs are pivotal in PDAC as they trigger both pro-inflammatory pathways that create a favorable tumor microenvironment and pathways leading to the production of immunosuppressive cytokines. Utilizing TLR agonists and antagonists in cancer therapy holds promise for enhancing survival rates.
Chen Z. et al. [33]	Association of the Microbiota and Pancreatic Cancer: Opportunities and Limitations	Review	2022	The interaction between pancreatic cancer and the microbiota involves both tumor-promoting and antitumor effects, showing changes in the “immune microsystem” within the tumor microenvironment. However, the great diversity of the microbiota and its multiple interactions with human factors such as age, sex, immune function, diet, climate, and geography pose significant challenges to harnessing the microbiota as an accurate treatment.
Rolfo C. et al. [34]	Applications and clinical trial landscape using Toll-like receptor agonists to reduce the toll of cancer	Review	2023	TLR agonists are promising for cancer treatment, particularly TLR7 and TLR9 agonists, either as a monotherapy or in combination with immune checkpoint inhibitors. While the most common side effects include several symptoms, these investigational TLR agonists, like CV8102 and tilsotolimod, do not worsen the toxicity of immune checkpoint inhibitors in combination regimens, which sets them apart from the discontinued TLR agonists with increased risk of adverse events.
Evans A. et al. [35]	The role of inflammatory cells in fostering pancreatic cancer cell growth and invasion	Mini Review	2012	Tumors can evade immune surveillance. The transition from an antitumor immune response to immune tolerance in the development of intraductal papillary mucinous neoplasm suggests the tumor-promoting roles of inflammatory cells, including immune suppression, angiogenesis, and metastasis, and highlights the need for further research to fully understand these roles in PDAC.
Jiang Z. et al. [36]	Functions and clinical applications of exosomes in pancreatic cancer	Review	2022	The use of exosomes as biomarkers for pancreatic cancer and their potential applications in cancer treatment are discussed, offering insights into the development of diagnostic tools and treatment strategies. However, challenges such as technical complexities, early stage clinical trials, and insufficient mechanistic research need to be addressed for effective clinical implementation.
Pergamo M. et al. [37]	Myeloid-derived suppressor cells and their role in pancreatic cancer	Review	2017	MDSCs play a crucial role in balancing immunogenic and tolerogenic signals by employing various mechanisms of immunosuppression. While targeting MDSCs has shown limited success to date, they represent a potential avenue for new immunotherapies that may prove effective in combating pancreatic cancer.
Yako Y.Y. et al. [38]	Cytokines as biomarkers of pancreatic ductal adenocarcinoma: a systematic review	Systematic Review	2016	Several studies consistently reported increased concentrations of IL-1β, IL-6, IL-8, VEGF, TGF, and IL-10 in PDAC patients, but their diagnostic performance has not been extensively tested, and they require validation in different study populations. These cytokines were associated with the severity of PDAC, suggesting a potential role as prognostic biomarkers, but further clinical evaluations are necessary to establish their clinical value for diagnostic, prognostic, or predictive purposes.
Li Y. et al. [39]	The Interplay Between Inflammation and Stromal Components in Pancreatic Cancer	Review	2022	The interaction between pancreatic cancer cells, stromal cells, and cytokines creates an inflammatory and immunosuppressive microenvironment, influencing various aspects of pancreatic cancer. Manipulating cytokine pathways holds promise for pancreatic cancer treatment, but challenges exist in translating animal model findings to human cancer. Single-cell sequencing technologies are aiding in defining the diversity and precise roles of stromal components in the tumor microenvironment, potentially revolutionizing personalized therapeutic approaches in the future.
Myo Min K.K. et al. [40]	Overcoming the Fibrotic Fortress in Pancreatic Ductal Adenocarcinoma: Challenges and Opportunities	Review	2023	PDAC presents a persistently low 5-year survival rate and limited benefits from conventional cancer treatments, including immunotherapy. A major challenge in PDAC therapy is effectively delivering drugs to the tumor due to the dense and complex tumor microenvironment, but renewed efforts in understanding and targeting this microenvironment offer hope for improved treatment efficacy.
Falcomatà C. et al. [41]	Context-Specific Determinants of the Immunosuppressive Tumor Microenvironment in Pancreatic Cancer	Review	2023	PDAC exhibits significant diversity, but a common feature is immunosuppression, limiting the effectiveness of immunotherapies. Understanding the distinct immunosuppressive niches and mechanisms in different PDAC subtypes is crucial, and advances in mouse modeling and high-throughput technologies can provide insights for the development of combinatorial immunomodulatory therapies to overcome these barriers.
Hawa Z. et al. [42]	The miRacle in Pancreatic Cancer by miRNAs: Tiny Angels or Devils in Disease Progression	Review	2016	MicroRNAs play a vital role in regulating gene expression, impacting various aspects of pancreatic cancer progression. These miRNAs have diagnostic and prognostic potential and could be considered as therapeutic tools, though addressing carrier-induced toxicity and further understanding that their downstream targets are crucial for their effective clinical application in combating pancreatic cancer.
Sato H. et al. [43]	Pancreatic Cancer Research beyond DNA Mutations	Review	2022	The early diagnosis of PDAC is crucial for improving patient survival. Understanding intercellular communication within the tumor microenvironment at the single-cell level may pave the way for highly individualized therapies, offering innovative treatments for advanced stages of the disease. Additionally, emerging diagnostic methods like VOC analysis hold potential for enhancing PDAC monitoring and may provide insights beyond DNA mutations, offering new avenues for addressing challenging cancers like PDAC.

Abbreviations: IL, interleukin; KRAS, Kirsten rat sarcoma virus: MDSC, myeloid-derived suppressor cells; miRNA, micro-RNA; PDAC, pancreatic ductal adenocarcinoma; TGF, tumor growth factor; TIL, tissue infiltrating lymphocytes; TNFα, tumor necrosis factor alpha; TLR, Toll-like receptor; VEGF, vascular endothelial growth factor; VOC, volatile organic compound.

**Table 2:** Summary of characteristics of included studies on PDAC and gut microbiota in most recent reviews, systematic reviews, and meta-analyses.

Author	Title	Type of Paper	Date	Finding
Ertz-Archambault N. et al. [7]	Microbiome and pancreatic cancer: a comprehensive topic review of literature	Review	2017	The connection between dysbiosis and pancreatic cancer, a disease known for its poor prognosis due to its late detection and resistance to treatment, is controversial. Mouse models indicate that altering the commensal microbiome can influence how tumors respond to chemotherapy in different cancer types, showing potential for improved survival and reduced cachexia in PDAC patients, offering insights into early screening biomarkers and novel therapeutic approaches.
Panthangi V. et al. [44]	Association Between Helicobacter pylori Infection and the Risk of Pancreatic Cancer: A Systematic Review Based on Observational Studies	Systematic Reviews and Meta-Analysis	2022	Few studies demonstrated a significant association between *H. pylori* infection and pancreatic cancer risk, primarily within European and Asian populations, with only one involving North Americans, indicating a weak association that does not provide conclusive evidence of *H. pylori’s* role in pancreatic cancer.
Pfisterer N. et al. [45]	The Microbiome in PDAC-Vantage Point for Future Therapies?	Review	2022	PDAC exhibits its own unique microbiome, influencing cancer development, treatment response, and patient prognosis. While the microbiome presents potential as a diagnostic and therapeutic tool in PDAC, inconsistencies in microbial composition require stringent decontamination procedures. Promising approaches, such as bacteriophages and fecal microbiome transplants, are still largely theoretical, and clinical trials are exploring the microbiome’s role as a biomarker for prognosis and surgical outcomes in PDAC.
Zhang C.Y. et al. [46]	Clinical diagnosis and management of pancreatic cancer: Markers, molecular mechanisms, and treatment options	Review	2022	Despite the approval of some therapies, overall survival rates remain poor, highlighting the need for novel immunotherapies, combined treatments, and advanced delivery methods to improve outcomes and combat drug resistance in PDAC patients, underscoring the importance of further clinical trials to evaluate these approaches.
Herremans, K.M. et al. [47]	The oral microbiome, pancreatic cancer and human diversity in the age of precision medicine	Review	2022	The oral microbiome, which changes in pancreatic cancer patients even before disease onset, can potentially serve as a noninvasive screening method to identify those at higher risk of developing cancer. Additionally, analysis of the oral microbiome could help guide treatment choices for patients and offer potential therapeutic options through microbial modification.
Sobocki B.K. et al. [48]	Pancreatic cancer and gut microbiome-related aspects: a comprehensive review and dietary recommendations	Review	2021	The connection between the gut microbiota and pancreatic cancer reveals that alterations in the gut microbiota can influence the development of pancreatic cancer. Methods like prebiotics, probiotics, next-generation probiotics, synbiotics, and fecal microbiota transplantation have the potential to be used as therapeutic strategies for pancreatic cancer.

Abbreviations: PDAC, pancreatic ductal adenocarcinoma.

**Table 3:** Summary of characteristics of included studies on PDAC and nutrition in most recent reviews, systematic reviews, and meta-analyses.

Author	Title	Type of Paper	Date	Finding
Emanuel A. et al. [49]	Nutritional Interventions in Pancreatic Cancer: A Systematic Review	Systematic Review	2022	The nutritional management of cachexia, malnutrition, and weight loss in pancreatic cancer is investigated. The findings suggest that enteral nutrition can have positive effects on various aspects, while dietary supplements enriched with omega-3 fatty acids appear to help maintain or increase body weight and lean body mass.
Cañamares-Orbís P. et al. [50]	Nutritional Support in Pancreatic Diseases	Review	2022	Patients with pancreatic diseases often face malnutrition, which is significant in chronic pancreatitis. Early diagnosis is key, as malnutrition can be due to both systemic factors related to the disease and specific factors like pancreatic enzyme insufficiency. Nutritional assessments, PERT, and personalized nutrition strategies are crucial in managing these conditions, and nutritional support can improve life quality and treatment tolerance, especially in pancreatic cancer.
Kasvis P. et al. [51]	Diet and Exercise Interventions in Patients With Pancreatic Cancer: A Scoping Review	Review	2021	This review evaluates the research gaps in dietary and/or exercise interventions previously studied in outpatients with pancreatic cancer.
Cortez N.E. et al. [52]	Ketogenic Diets in Pancreatic Cancer and Associated Cachexia: Cellular Mechanisms and Clinical Perspectives	Review	2021	This review highlights the impact of the ketogenic diet (KD) in PDAC treatment and cachexia, reporting the potential anticancer and anti-cachexia effects of KD.
Veronese N. et al. [53]	Dietary fiber and health outcomes: an umbrella review of systematic reviews and meta-analyses	Umbrella Review	2018	A comprehensive overview on the associations between dietary fiber intake and inflammation, cancer and cardiovascular disease.
Guo Z. et al. [54]	Dietary inflammatory index and pancreatic cancer risk: a systematic review and dose–response meta-analysis	Systematic Review and Meta-Analysis	2021	The review reports the link between pancreatic cancer risk and the Dietary Inflammatory Index (DII) score, which assesses the inflammatory component of foods. A high DII score corresponds to eating habits with high inflammatory characteristics.
Jayedi A. et al. [55]	Dietary Inflammatory Index and Site-Specific Cancer Risk: A Systematic Review and Dose–response Meta-Analysis	Review	2018	This review reports the associations of the Dietary Inflammatory Index (DII) and site-specific cancer risk.
Lee H.J. et al. [56]	Role of omega-3 polyunsaturated fatty acids in preventing gastrointestinal cancers: current status and future perspectives	Review	2018	Potent natural products like n-3 PUFAs, known for their cancer-preventive properties, have shown potential in altering cancer cell properties and the tumor microenvironment, but more research, including clinical trials, is needed to determine their optimal use and efficacy in cancer prevention.
Liu Y. et al. [57]	Vitamin intake and pancreatic cancer risk reduction: A meta-analysis of observational studies	Systematic Review and Meta-Analysis	2018	The intake of vitamins, especially vitamin D and vitamin B12, was associated with a moderate reduction in the risk of pancreatic cancer.
Zhang Z. et al. [58]	Therapeutic Intervention in Cancer by Isoliquiritigenin from Licorice: A Natural Antioxidant and Redox Regulator	Review	2022	Chemoresistance and poor efficacy of combination therapies with drugs like gemcitabine have been significant challenges in treating pancreatic cancer. Targeting autophagy, specifically inhibiting it, may be a promising strategy to improve chemotherapy, and isoflavonoids from herbal sources like ISL could help overcome chemoresistance, potentially enhancing the survival and quality of life for patients.
Asgharian P. et al. [59]	Quercetin Impact in Pancreatic Cancer: An Overview on Its Therapeutic Effects	Review	2021	This review highlights the effects of quercetin on pancreatic cancer. It inhibits c-Myc and TGF-α expression and promotes autophagy and cancer cell apoptosis
Koltai T. et al. [60]	Role of Silymarin in Cancer Treatment: Facts, Hypotheses, and Questions	Review	2021	Silymarin exhibits significant antitumoral effects, and clinical trials are needed to determine its clinical applications, particularly its dual effects. The use of high doses, as well as modified pharmaceutical forms, is recommended due to silymarin’s low absorption, and it has potential in cancer treatment and prevention.
Hu X. et al. [61]	New possible silver lining for pancreatic cancer therapy: Hydrogen sulfide and its donors	Review	2021	H_2_S donors have shown potential as therapeutic agents in pancreatic cancer, and the development of dual H_2_S and NO donor drugs is underway to address cell proliferation, apoptosis, and cell cycle modulation. However, challenges such as targeted delivery, H_2_S concentration maintenance, and clarification of the dual H_2_S/NO donor’s mechanisms need further attention.
Yang, Q. et al. [62]	Potential Roles of the Gut Microbiota in Pancreatic Carcinogenesis and Therapeutics.	Review	2022	The gut microbiota is crucial in pancreatic cancer regulation, offering insights into mechanisms, risk prediction, diagnosis, and prognosis. This research highlights the potential of probiotics in combination with chemotherapy and immunotherapy for pancreas cancer treatment, emphasizing the importance of investigating cancer-related microbiome approaches and personalized treatments to enhance patient outcomes.
Ibrahim, M.O. et al. [63]	What Dietary Patterns and Nutrients Are Associated with Pancreatic Cancer? Literature Review.	Review	2023	The high consumption of sugar, fructose, and fat may elevate pancreas cancer risk, while dietary fiber, fat-soluble vitamins, water-soluble vitamins, and minerals may have a protective effect. Additionally, dietary patterns rich in plant-based foods, antioxidants, and polyphenols appear to reduce cancer risk, while patterns high in red and processed meats, sugar, and sugar-sweetened soft drinks are associated with an increased risk.

Abbreviations: DII, Dietary Inflammatory Index; HDAC, histone deacetylase inhibitors; H_2_S, hydrogen sulfide; KD, ketogenic diet; NO, nitric oxide; PDAC, pancreatic ductal adenocarcinoma; TGF-α, transforming growth factor alpha; ω-3, omega-3 polyunsaturated fatty acids.

## Data Availability

Not applicable.
